# Pregnancy of patients with idiopathic thrombocytopenic purpura: maternal and neonatal outcomes

**DOI:** 10.4274/jtgga.galenos.2019.2019.0078

**Published:** 2020-06-08

**Authors:** Hakan Kalaycı, Gülşen Doğan Durdağ, Şafak Yılmaz Baran, Seda Yüksel Şimşek, Songül Alemdaroğlu, Serdinç Özdoğan, Esra Bulgan Kılıçdağ

**Affiliations:** 1 Department of Obstetrics and Gynecology, Başkent University Faculty of Medicine, Adana Application and Research Hospital, Adana, Turkey

**Keywords:** Idiopathic thrombocytopenic purpura, neonatal thrombocytopenia, pregnancy, thrombocytopenia

## Abstract

**Objective:**

Thrombocytopenia occurs in 7% of pregnant women. Along with other causes, idiopathic thrombocytopenic purpura (ITP), which is an autoimmune disease with autoantibodies causing platelet destruction, must be considered in the differential diagnosis. Antiplatelet antibodies can cross the placenta and cause thrombocytopenia in the newborn. The aim of our study was to assess the management of ITP in pregnancy, and to investigate neonatal outcomes.

**Material and Methods:**

This retrospective study was conducted in a tertiary center including 89 pregnant patients with ITP followed between October 2011 and January 2018. Patients were evaluated in two groups according to diagnoses of ITP and chronic ITP. Age, obstetric history, ITP diagnosis, and follow-up period, presence of splenectomy, platelet count during pregnancy and after birth, treatment during pregnancy, route of delivery, weight and platelet count of newborn, sign of hemorrhage, and fetal congenital anomaly were assessed

**Results:**

Considering the ITP and chronic ITP groups, no significant difference was seen with respect to parity, timing of delivery, preoperative and postoperative platelet counts, and hemoglobin values. Route of delivery, birth weight, APGAR scores, newborn platelet count, and congenital anomaly rates were also similar. The timing of treatment was different because patients whose diagnoses were established during pregnancy were mostly treated for preparation of delivery. Treatment modalities were similar.

**Conclusion:**

Probability of severe thrombocytopenia at delivery is higher in patients with ITP who are diagnosed during pregnancy when compared with patients who received prepregnancy diagnoses. ITP is an important disease for both the mother and newborn. Patients should be followed closely in cooperation with the hematology department.

## Introduction

Thrombocytopenia, which is defined as a platelet count being less than 150x10^3^/µL, occurs in approximately 7% of pregnant women (1). Various etiologies can cause thrombocytopenia during pregnancy. The most commonly seen, gestational thrombocytopenia and idiopathic thrombocytopenic purpura (ITP), are both diagnoses of exclusion of other pathologies necessitating different treatment strategies. These pathologies include preeclampsia; HELLP syndrome characterized by hemolysis, elevated liver enzymes and low platelet count; sepsis; disseminated intravascular coagulation; autoimmune diseases such as systemic lupus erythematosus, thrombotic thrombocytopenic purpura; microangiopathies such as hemolytic uremic syndrome; hematologic malignancies; and drug-induced thrombocytopenia (2,3,4).

Gestational thrombocytopenia, which usually occurs in the mid-second to third trimester and which is a mild form with platelet counts more than 70x10^3^/µL, constitutes 70-80% of cases (1,5). ITP, which is an autoimmune disease with autoantibodies against the platelet membrane causing platelet destruction in the reticuloendothelial system, must be considered in the differential diagnosis. The incidence of ITP at pregnancy is 1-2/1000 and it forms 3-5% of thrombocytopenias encountered during pregnancy, though it is the most common cause of thrombocytopenias in early pregnancy and can cause severe thrombocytopenia (2,5,6). Patients may be asymptomatic or may have antenatal bleeding necessitating treatment. Thrombocytopenia may be first diagnosed during pregnancy or the diagnosis of ITP may have been established before pregnancy. ITP lasting more than 6 months is called chronic ITP (1). Splenectomy may be performed due to resistance to treatment. Time of diagnosis, severity, and accompanying factors must be considered for the differential diagnosis of thrombocytopenia. The onset time of ITP can affect pregnancy and delivery complications, and clinical conditions of the newborn such as thrombocytopenia, petechiae, and intracranial hemorrhage (1).

The aim of our study was to assess the follow-up and treatment of ITP and chronic ITP during pregnancy, along with neonatal outcomes. Proper diagnoses and management in the antenatal period will reduce complications.

## Material and Methods

This retrospective study was conducted at a university hospital, including pregnant patients with ITP. A total of 89 patients followed between October 2011 and January 2018 in our center with ICD diagnose codes of pregnancy Z33, Z34.8, Z35.8, Z35.9 and concurrent D69.3 ITP or D69.6 thrombocytopenia codes were included. The records of patients were investigated, and phone calls with patients were used to collect missing data. Other causes of thrombocytopenia were excluded.

Age, obstetric and medical history, time of ITP diagnosis and duration of follow-up period, presence of splenectomy, platelet counts in early and late pregnancy and after birth, complete blood count and biochemistry parameters, treatment during pregnancy, route of delivery and anesthesia, need for thrombocyte or erythrocyte replacement, gestational week at time of birth, weight, APGAR scores and platelet count of newborn, signs of fetal hemorrhage, and fetal congenital anomalies were assessed.

This study was approved by Institutional Review Board and Başkent University Ethics Committee (approval number: KA 18/70). Informed consent was obtained.

### Statistical Analysis

The SPSS 23.0 program was used for statistical analysis. Categorical measurements were assessed as number and percentage, continuous measurements are summarized as mean and standard deviation. The chi-square or Fisher’s exact test statistics were used to compare categorical variables. To compare continuous variables between the groups, ranges were assessed, ANOVA or Student’s t-test were used in dual groups for variables in a parametric range. P<0.05 was considered significant for all tests.

## Results

The mean age of the 89 patients included in the study was 30 years, and the mean age at ITP diagnosis was 28 years. The mean gestational week at birth was 37 weeks and 5 days, the mean birth weight was 3073 g, and the mean hemoglobin preoperatively and postoperatively was 11.7 g/dL and 10.4 g/dL, respectively. The mean platelet count of the patients was 98x10^3^/µL, ranging between 19x10^3^/µL and 622x10^3^/µL. The mean platelet count of the newborns was 226x10^3^/µL. The mean parity of patients was 2. Chronic ITP was diagnosed in 36% of the patients. Time of diagnosis was in the first trimester in 11% of patients, the second trimester in 18% of patients, and in the third trimester in 34% of patients. The rate of women who smoked was 6.7%. Cesarean section was performed for 75% of patients due to previous cesarean or other obstetric indications, and 11% of these patients were underwent neuraxial anesthesia. Antenatal bleeding was not encountered in 84.3% of patients, 10.3% had vaginal bleeding, and 4.3% had bleeding of other sites not related to pregnancy. Of all the patients, 42.7% required treatment, 28.9% of which was for delivery preparation. Treatment was administered in the third trimester in 44.7% of cases, and 26.3% received treatment in first or second trimester.

The treatment modality was steroids for 12.4% of patients, platelet transfusion for 9.0%, steroid and platelet transfusion for 7.9%, and steroid and platelet transfusion and intravenous immunoglobulin (IVIG) for 13.5% of patients. No treatment was needed for 57.3% of the patients. Erythrocyte transfusion beyond other treatments was administered to 11 patients. Splenectomy was performed in five (5.6%) patients.

Of the 89 pregnancies, one resulted in missed abortus, one resulted in termination of pregnancy, one ended with intrauterine exitus at the 26^th^ week, and delivery data of five patients could not be obtained due to delivery at other centers. Data of 81 newborns were assessed. Neonatal intensive care unit (NICU) admission was needed for 19.7% of the newborns. Seven of the 81 (8.6%) newborn babies had thrombocytopenia. Babies without thrombocytopenia in the first examination were not checked again in terms of platelet count. Babies with a platelet count <30x10^3^/µL were hospitalized and their platelet counts were checked daily, and platelet counts >50x10^3^/µL were checked every 2-3 days.

When patients were classified into two groups as ITP and chronic ITP, 57 patients were in the ITP group and 32 patients were in the chronic ITP group. No significant difference was seen between the two groups with respect to parity, timing of delivery, birth weight, preoperative and postoperative platelet counts and hemoglobin values, newborn platelet counts, 1^st^ and 5^th^ minute APGAR scores, and NICU admission. The times of diagnosis were significantly different between the two groups, the mean age being 30.3 years for patients with ITP and 23.8 years for patients with chronic ITP (p<0.001) (Table 1). The smoking rate was significantly higher in the chronic ITP group (p=0.001).

Route of delivery, antenatal bleeding, and congenital anomaly rates were similar. There were three congenital anomalies leading to neonatal death in the chronic ITP group; aortic coarctation - ventricular septal defect - patent ductus arteriosus - pulmonary hypertension in the first baby, transposition of the great arteries in the second baby, and patent ductus arteriosus in the third baby. The latter baby also had thrombocytopenia, and had intraventricular and subdural hemorrhage. None of the other babies had intracranial hemorrhage. In the ITP group, one pregnancy was terminated due to a severe skeleton deformity, one baby had hydroureteronephrosis, and two babies had hypospadias. Furthermore, one pregnancy resulted in intrauterine exitus at the 26^th^ week in the ITP group, and there was one missed abortus in the chronic ITP group.

Of all the patients, low platelet counts were found in 7 of the newborn babies, 4 of whom were in the ITP group with platelet count between 51-66x10^3^/µL, and 3 babies whose platelet counts were <30x10^3^/µL were in the chronic ITP group. These babies underwent transfontanel and abdominal ultrasound imaging, and no hemorrhage was found except in the above-mentioned baby who had patent ductus arteriosus.

The timing of treatment was different between the groups (p=0.034) (Table 2). Patients whose diagnoses were established during pregnancy were mostly treated for preparation of delivery. Treatment modalities were similar (Table 3). Erythrocyte transfusion rates were also similar.

All patients who had splenectomy were in the chronic ITP group, and they underwent splenectomy surgery before or after pregnancy; therefore, none of our patients required such surgery for treatment during pregnancy.

## Discussion

The differential diagnosis of thrombocytopenia in pregnancy may be difficult. The onset time of thrombocytopenia, and its severity and relationship with other abnormal clinical conditions direct the diagnosis. Gestational thrombocytopenia and ITP diagnosed after the second trimester of pregnancy may be especially difficult to differentiate. However, ITP causes more severe thrombocytopenia and it may necessitate treatment at the beginning of pregnancy or in the proceeding weeks.

Steroid and IVIG are the most commonly used treatments. However, timing of treatment may affect potential adverse effects. Steroids, which are used as the first step in treatment, may cause fetal anomalies when used in early pregnancy. Furthermore, it must be kept in mind that steroids may increase complications such as hypertension, gestational diabetes, and premature labor (2). When the thrombocyte response to steroids is insufficient or the adverse effects are intolerable, IVIG treatment can be used alone or in combination with low- dose steroids (4). Sun et al. (7), who compared the effectiveness of steroid and IVIG treatments in their retrospective study with 235 pregnant patients, found no significant difference between the groups with respect to maternal platelet count and newborn results. Wang et al. (3) also declared similar treatment efficacy. Other drugs such as mycophenolate mofetil, immunosuppressants such as azathioprine, and thrombopoietin receptor agonists can also be used for treatment (4,8). Treatment choice is determines based on efficacy, toxicity, and the cost of the drugs. In our study, 57.3% of the patients did not need treatment, 30.3% were given platelet transfusion alone or in combination with other treatments.

The ITP and chronic ITP groups were similar with respect to all treatment modalities used. Platelet transfusion is mostly effective in emergency cases when there is little time to wait for the results of other treatments. Gernsheimer et al. (4), reported 5-18.9% platelet transfusion rates before delivery in patients with ITP. Some of our patients were followed up in other hospitals and were canalized for delivery at our hospital as a tertiary center. This situation can explain the higher rates of platelet transfusion when compared with previous studies.

Resistance to treatment or severe toxicity may necessitate splenectomy for remission of the patient. This procedure can be performed in the second trimester; however, it is emphasized that transplacental crossing of maternal antibodies and the risk of neonatal thrombocytopenia is not affected by splenectomy (4). None of our patients necessitated splenectomy for treatment during pregnancy.

Wyszynski et al. (1) conducted a study with 446 pregnant patients with ITP, and they concluded that fetal demise, premature delivery, and congenital anomaly risk were higher in patients diagnosed as having ITP or chronic ITP before pregnancy when compared with patients diagnosed during pregnancy, and maternal ITP duration affected pregnancy significantly; however, other medical conditions of the mother and the medications used were not mentioned and their effects could not be evaluated. Subbaiah et al. (2), in their study with 30 pregnancies of 26 patients with ITP, found no increased rates of preterm delivery, low birth weight, still birth or neonatal death in pregnancies of patients with ITP. Moreover, they reported no significant difference between the platelet count in early pregnancy and before delivery. Of our patients, 64% were new diagnosed in pregnancy, and 36% had chronic ITP. When we compared these two groups, gestational week at delivery, birth weight, and congenital anomalies were similar. Also, in Subbaiah et al.’s (2) study, the probability of severe thrombocytopenia at delivery was shown to be higher in patients with ITP who were diagnosed during pregnancy when compared with patients who received their diagnosis before pregnancy. The difference in treatment time in our study is consistent with this result.

As stated previously, route of delivery as normal vaginal birth or cesarean section does not affect hemorrhagic complications in patients with ITP, and the decision must be led due to obstetric indications (5,6). Gernsheimer et al. (4) reported that treatment would usually not be necessary in the absence of bleeding symptoms or when the number of platelets was >30x10^3^/µL in pregnant women with ITP. Won et al. (6) also suggested that the number of platelets of mother should be >30x10^3^/µL during pregnancy and >50x10^3^/µL near delivery. Furthermore, more than 50x10^3^/µL platelets for normal vaginal birth or cesarean section, and more than 80x10^3^/µL platelets for neuraxial anesthesia was requested. It was emphasized that for asymptomatic patients, treatment might not be necessary until delivery if the platelet count was above 20x10^3^/µL; however, these patients should be followed closely. Lee et al. (9) also stated little risk of epidural hematoma related to neuraxial anesthesia for platelet counts >70x10^3^/µL.

In our own clinical practice, neuraxial anesthesia is preferred for patients with platelet numbers above 100x10^3^/µL. Besides, in our center, the recommendation of the hematology department is mostly close monitoring of platelet count and follow-up without treatment as long as the platelet count is >30x10^3^/µL and the patient is asymptomatic. Steroid treatment is recommended in the event of decline in the platelet count and when the patient is symptomatic; >70x10^3^/µL platelets are suggested for delivery, and close follow-up of the newborn in terms of immune thrombocytopenia is recommended. Also, the platelet counts must be evaluated using peripheral blood smears before treatment.

Antiplatelet antibodies can cross the placenta and cause thrombocytopenia in the newborn. Wounds on the face and scalp; petechiae; cleft palate; intraventricular hemorrhage and hydrocephalus; and cardiac anomalies such as atrial septal defect, ventricular septal defect, and patent ductus arteriosus may be seen in the newborn. Preterm labor or low birth weight may be encountered both in patients with ITP and chronic ITP. Maternal ITP resistant to splenectomy was reported to be related to higher rates of intracranial hemorrhage in the newborn (1). Furthermore, it was shown in several studies that maternal platelet count at delivery was not related to the platelet count of the newborn and maternal treatment did not affect the newborn platelet count. It was emphasized that risk factors for neonatal thrombocytopenia were maternal history of splenectomy, maternal platelet count less than 50x10^3^/µL in pregnancy, and neonatal thrombocytopenia in the previous pregnancies of the mother (3,5). Among our patients, there were seven newborns with thrombocytopenia, and platelet counts were <50x10^3^/µL in three of the mothers in this subgroup, besides, one of the mothers had splenectomy; therefore, these numbers may not be sufficient to draw a conclusion on the risk factors of neonatal thrombocytopenia.

Fujimura et al. (10) reported neonatal thrombocytopenia as 9-15%; however, they denoted that risk of fetal intracranial hemorrhage was very low. Loustau et al. (11), reported a rate of 8.3% severe thrombocytopenia without hemorrhagic complications in the newborn. The neonatal thrombocytopenia rate was 8.6% in our study, and we found no significant difference between the ITP and chronic ITP groups in this aspect. Gernsheimer et al. (4) reported that the neonatal platelet count was not needed to be repeated if it was normal in the first examination. However, for newborns with thrombocytopenia, the thrombocyte count should be checked daily and cranial ultrasound should be performed when the platelet count is <50.000. Our practice is consistent with these implementations.

### Study Limitations

The limitations of our study are its retrospective nature and the missing data. The obstetric records of the referred patients were based on patient anamnesis. Also, some of the patients were admitted to other centers for delivery, which led to incomplete newborn data.

## Conclusion

ITP diagnosed before or during pregnancy is important for both the mother and the newborn. The risk of severe thrombocytopenia at delivery is higher in ITP when compared with chronic ITP. Patients should be followed closely at a tertiary center in cooperation with a hematology department. The probability of hemorrhage during pregnancy, and preparation for either normal vaginal birth or cesarean section must all be considered and the platelet count must be kept stable. The newborn’s platelet count must also be closely followed in terms of immune thrombocytopenia.

## Figures and Tables

**Table 1:**
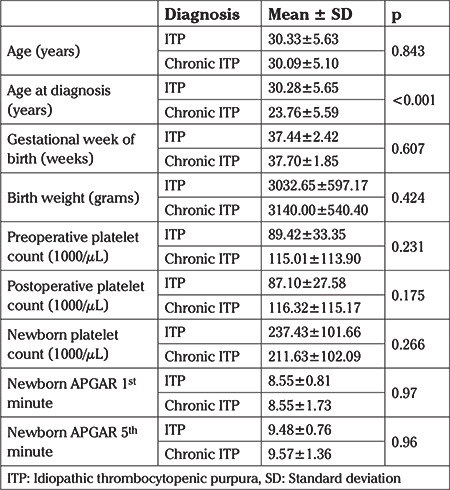
Comparison of mean values of patients with idiopathic thrombocytopenic purpura (ITP) and chronic ITP

**Table 2:**
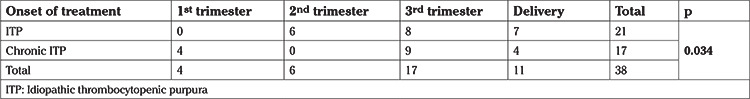
Timing of treatment for the idiopathic thrombocytopenic purpura (ITP) and chronic ITP groups

**Table 3:**
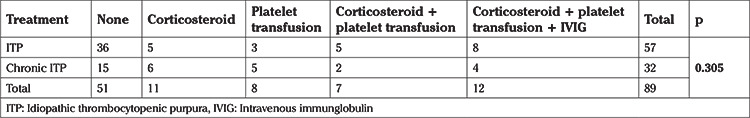
Treatment choice for idiopathic thrombocytopenic purpura (ITP) and chronic ITP groups
